# Quality control inclinical diagnostic laboratories in remote and rural areas in Africa

**Published:** 2012-12-09

**Authors:** Subhash C Arya, Nirmala Agarwal

**Affiliations:** 1 Sant Parmanand Hospital, Delhi, India

**Keywords:** Quality control, non-academic centers, rural areas, laboratory infrastructure

## Abstract

The authors propose a way to improve the quality of results generated through the fragile clinical laboratory infrastructure in resource poor counties in Africa and elsewhere.

## To the editors of the Pan African Medical Journal

We have a proposal to improve the quality of results generated through the fragile clinical laboratory infrastructure in resource poor counties in Africa and elsewhere. In several urban areas and remote and rural locations, private clinical laboratories might be the only ones serving vast populations.In order to ensure better quality of patient care, urgent donor support, local capacity building, pathology and laboratory medicine training, supply of reagents and maintenance of existing instrumentation have been recommended [1]. There has been concern about the quality of results obtained in clinical research a laboratory in sub-Saharan Africa even in the National Institute of Health-funded laboratories [2].

We feel that the concept of quality control in clinical laboratories in Africa could be implemented in the existing laboratory systems. For example, a beginning could be made with a biochemical test for blood glucose level. Laboratories in remote locations, even if lacking in trained personnel or sophisticated instrumentation, and little hope of receiving external funding, could be encouraged to pick up blood samples from patients with a high or low glucose level to prepare its 20 aliquots. By testing such aliquots repeatedly, it would be possible to work out local mean glucose level and standard deviation (SD) [3]. If the results of this basic and most common test are consistently satisfactory, it can be presumed that the results of other investigations would also be valid.

In our opinion, rather than an outright purchase of laboratory analyzers for hematology or clinical chemistry investigations, their procurement on rent-reagent basis would be useful in all laboratories in resource-poor countries in Africa. In such an arrangement between diagnostic companies and laboratories, an analyzer would be placed in the laboratory in exchange for the guaranteed purchase of reagents over time. Alternatively, the laboratory would pay a specified amount per test run. Such contracts would allow laboratories to avoid capital outlay on the purchase of analyzers. Even contracts incorporating service maintenance are also popular [4]. Consequently, the supplier of analyzer and reagents would ensure constant maintenance of equipment and supplies of consumables.

A regular in-house scrutiny of the stocks of laboratory reagents in the laboratory premises or a storage depot in a neighbouring location would mitigate, if not eliminate, their sudden depletion. Introduction of a weekly review of stocks available in the laboratory and the hospital warehouse, active participation of laboratory personnel, warehouse manager, and purchase manger will ensure regular availability of required laboratory supplies [5].

## Conclusion

In conclusion, improvement in the quality of laboratory results would eventually be beneficialto the people community in general in Africa, through active, phased participation of non-research, non-academic laboratories in big cities, as well as in rural areas. While awaiting an upgrade of laboratories through harmonized international donor support1, each laboratory should support an in-house scrutiny of their results at least with the most common investigations carried out by them [3].
